# Smoking-related psychosocial beliefs and justifications among smokers in India: Findings from Tobacco Control Policy (TCP) India Surveys

**DOI:** 10.1186/s12889-022-14112-w

**Published:** 2022-09-13

**Authors:** Anupreet K. Sidhu, Mangesh S. Pednekar, Geoffrey T. Fong, Prakash C. Gupta, Anne C. K. Quah, Jennifer Unger, Steve Sussman, Neeraj Sood, Heather Wipfli, Thomas Valente

**Affiliations:** 1 Tobacco Center of Regulatory Science and Department of Psychiatry, Perelman School of Medicine, University of Pennsylvania, Philadelphia, PA USA; 2 Healis-Sekhsaria Institute for Public Health, Mumbai, India; 3 Department of Psychology, University of Waterloo, Waterloo, ON Canada; 4 School of Public Health Sciences, University of Waterloo, Waterloo, ON Canada; 5 Ontario Institute for Cancer Research, Toronto, ON Canada; 6 Department of Population and Public Health Sciences, Keck School of Medicine, University of Southern California, Los Angeles, CA USA; 7 Sol Price School of Public Policy and Schaeffer Center, University of Southern California, Los Angeles, CA USA

**Keywords:** Smoking, Cognitive dissonance, Psychosocial beliefs, Quitting, Tobacco use

## Abstract

**Background:**

Previous research in high-income countries (HICs) has shown that smokers reduce their cognitive dissonance through two types of justifications over time: risk minimizing and functional beliefs. To date, however, the relationship between these justifications and smoking behaviors over time has limited evidence from low- and middle-income countries. This study examines these of justifications and their relation to quitting behavior and intentions among smoking tobacco users in India.

**Methods:**

The data are from the Tobacco Control Policy (TCP) India Survey, a prospective cohort of nationally representative sample of tobacco users. The respondents include smoked tobacco (cigarettes and bidi) users (*n* = 1112) who participated in both Wave 1 (W1; 2010–2011) and Wave 2 (W2; 2012–2013) surveys. Key measures include questions about psychosocial beliefs such as functional beliefs (e.g., smoking calms you down when you are stressed or upset) and risk-minimizing beliefs (e.g., the medical evidence that smoking is harmful is exaggerated) and quitting behavior and intentions at Wave 2.

**Findings:**

Of the 1112 smokers at W1, 78 (7.0%) had quit and 86 (7.8%) had intentions to quit at W2. Compared to W1, there was a significant increase in functional beliefs at W2 among smokers who transitioned to mixed use (using both smoking and smokeless tobacco) and a significant decrease among those who quit. At W2, smokers who quit held significantly lower levels of functional beliefs, than continuing smokers, and mixed users ((M = 2.96, 3.30, and 3.93, respectively, *p* < .05). In contrast, risk-minimizing beliefs did not change significantly between the two waves. Additionally, higher income and lower functional beliefs were significant predictors of quitting behavior at W2.

**Conclusion:**

These results suggest that smokers in India exhibit similar patterns of dissonance reduction as reported in studies from HICs: smokers who quit reduced their smoking justifications in the form of functional beliefs, not risk-minimizing beliefs. Smokers’ beliefs change in concordance with their smoking behavior and functional beliefs tend to play a significant role as compared to risk-minimizing beliefs. Tobacco control messaging and interventions can be framed to target these functional beliefs to facilitate quitting.

**Supplementary Information:**

The online version contains supplementary material available at 10.1186/s12889-022-14112-w.

## Background

In India, as in the vast majority of countries, tobacco use is a major public health concern. Despite widespread knowledge of tobacco use risks and harm, staggering numbers of smokers continue this deadly behavior. When people continue to smoke despite knowing the harms of smoking, it creates cognitive dissonance which is an aversive emotional state that leads to motivation to reduce that dissonance [1]. Because smoking is so addictive, reducing dissonance by changing behavior does not happen very often; therefore, smokers may resort to dissonance reduction by changing one’s dissonant beliefs [2].

Social psychological research demonstrates that because quitting is very difficult [3, 4], smokers may generate beliefs to justify their smoking [5]. Adult smokers who continue to use tobacco despite knowledge of harmful effects of smoking engage in dissonance reduction using justifications for continuing smoking. These justifications are also referred to as rationalizations, disengagement beliefs, or self-exempting beliefs [6–8]. These justifications have been characterized in a number of ways; some beliefs act as a shield for smokers, providing false reassurances, and enabling avoidance of thinking deeply about quitting [9]. Two types of beliefs are *functional beliefs*, which serve to highlight the perceived benefits of smoking, such as increased concentration, stress reduction, and *risk-minimizing beliefs*, which justify smoking by undermining the harms and negative health consequences of smoking [6, 9–14]. Multiple cross-sectional studies and longitudinal studies have found that high endorsements of pro-smoking beliefs are associated with lower quit intentions among smokers [4–6, 9, 10, 15, 16]. Additionally, previous research has shown associations between price promotions and functional beliefs in some HICs [17].

The vast majority of research on the interplay between smoking and dissonance reducing beliefs has been conducted in high-income Western countries. There are, however, some studies that have been conducted in Asian countries. An analysis of predictors of intentions to quit among smokers in Korea found no significant association of risk-minimizing beliefs (termed as self-exempting beliefs in the study) with intentions to quit smoking [18]. Higher smoking rationalizations were associated with lower intentions to quit among male smokers in China [19]. Another study from Southeast Asia found higher prevalence of rationalization (“You’ve got to die of something, so why not enjoy yourself and smoke”) among Malaysian smokers compared to Thai smokers, which may discourage cessation efforts in Malaysia with lower levels of intentions to quit [4]. The patterns of rationalizations and the association between regret and rationalization were different between Thailand and Malaysia; therefore, it is important to analyze smoking rationalizations and justifications in different countries to understand the belief systems and design counter-tobacco messaging accordingly.

There has been important research conducted on the role of functional and risk-minimizing beliefs to sustain smoking [6, 10]. These have been shown to be important as justifications for continued smoking over time, and they tend to reduce when a smoker quits and bounce back when a quitter relapses. This is indicative of the use of these beliefs to reduce the strong level of cognitive dissonance that arises when a smoker continues to smoke in the face of the knowledge that smoking is dangerous. In fact, a study by Fotuhi et al. [5] assessed if smokers adjusted their beliefs in patterns consistent with Cognitive Dissonance Theory [1] while determining the magnitude of belief change among smokers accompanying behavior change. The study found that smokers tend to rationalize their smoking behaviors and those beliefs change systematically with their smoking status.

Most studies that have examined cognitive dissonance and dissonance reduction among smokers have been conducted in high-income countries (HICs). Though some of these studies are from low- and middle-income countries, these studies were largely cross-sectional, which limits the ability to assess causal relationships between beliefs and quitting. This study aims to investigate the smoking related beliefs and their association with quitting behaviors among smokers in India, and to understand how the evidence stacks up in relation to what we know from HICs. The proposed study is designed to address the following aims: 1) To examine the pattern of functional and risk-minimizing beliefs (justifications) among smokers in India. 2) To assess changes in and associations of smoking justifications with quitting intentions and behavior over time. This study is among the first to examine the predictive value of two kinds of beliefs: functional beliefs and risk-minimizing beliefs and how they may predict future quitting among smokers in India.

## Methods

This study is a part of the larger Tobacco Control Policy (TCP) India Survey, a prospective cohort study of adult tobacco users (aged 15 +) and non-users from 4 Indian states: Bihar, Madhya Pradesh (MP), Maharashtra, and West Bengal (WB). Within each state, one major city represented an urban area and a surrounding area within 50 km outside the city represented a rural area. At Wave 1, the survey employed a stratified multistage cluster sampling design and was conducted between August 2010 and October 2011. Wave 2 was conducted in October 2011 to September 2013. The survey protocol and questionnaires were first developed in English followed by translation into the dominant languages of each state (Hindi in Bihar and MP, Marathi in Maharashtra, and Bengali in WB). At the end, respondents were debriefed, remunerated, and thanked for their time [20, 21]. Additional details on the construction of survey weights, household enumeration, selection criteria and response rates are available in TCP India Technical Reports [22, 23].

### Study sample

Data for this study were drawn from the TCP India Survey comprising 8940 participants and only baseline smoked tobacco users who participated at both waves of data collection were selected for analysis. Of the 8940 participants sampled at Wave 1, 1255 were smoked tobacco users. Of those 1255 smokers, 1112 were followed up at Wave 2 and reported their tobacco use status. The analytical sample of smokers had an 88.6% retention rate at Wave 2.

### Measures

#### Socio-demographic variables

Socio-demographic variables measured were age, sex, highest level of educational attainment, monthly household income and urban residence. Education was categorized into low, moderate, and high. Low education included illiterate, primary or middle school education; moderate included secondary school or Industrial Training Institute courses; and high included those who completed college and higher education. Similarly, income level was divided into low, moderate, and high. Low-income category included those earning less than 5000 INR per month; moderate income had those earning between 5000 and 15,000 INR per month and high income included those earning more than 15,000 INR.

#### Smoking tobacco user

A smoker was defined as anyone who said yes to either of the following questions: “Do you currently smoke cigarettes at least once a month?” or “Do you currently smoke bidis at least once a month?” (Yes/No/Don’t Know).

#### Tobacco use variables

The tobacco use variables were the use frequency, intention to quit smoking, and quit status.

##### Use frequency

The cigarette and bidi smoking frequency were measured by two different questions asking: “On average, how often do you smoke cigarettes?” and “On average, how often do you smoke bidis?” The response categories – “Less than once a week/Once a week/Twice a week/3–5 times a week/Every day or almost every day More than once a day” – were combined and reported as daily smoker (Every day or almost every day/More than once a day), less than daily smoker (Once a week/Twice a week/3–5 times a week), and less than weekly smoker (Less than once a week) for cigarette and bidi users separately.

##### Intention to quit

Intention to quit was measured by asking “Are you planning to quit smoking…” and the response categories were: “Within the next month/Within the next 6 months/Sometime in the future, beyond 6 months/Not planning to quit/Refused/Don’t know.” The responses were recoded as a dichotomous variable with any plans to quit as 1 or Yes and “Not planning to quit/Refused/Don’t know” as 0 or No.

##### Quitting

At Wave 2, all smokers from Wave 1 were asked whether they were still smoking. Those who indicated that they had completely quit smoking were categorized as 1 (having quit) and those who continued smoking or transitioned to mixed use were coded as 0 (continuing smoking).

#### Psychosocial beliefs

Functional beliefs were assessed using three statements: (F1) You enjoy smoking too much to give it up, (F2) Smoking calms you down when you are stressed or upset, and (F3) Smoking is an important part of your life. Risk-minimizing beliefs were assessed using the following three statements: (R1) The medical evidence that smoking is harmful is exaggerated, (R2) Everybody has got to die of something, so why not enjoy yourself and smoke, and (R3) Smoking is no more risky than lots of other things that people do. These psychosocial beliefs were measured on a five-point Likert-scale ranging from Strongly agree [5] to Strongly disagree [1]. These beliefs were also dichotomized for frequency analysis where Strongly agree/Agree were coded as 1 (having a belief) and the Neither agree nor disagree/Disagree/Strongly Disagree were coded as 0 (NOT having a belief).

### Data analysis

Analyses were conducted using STATA/SE 17. Univariate statistics were used to categorize the sample and bivariate statistics such as paired t-tests were conducted to analyze the difference between justifications and smoking status between two waves. Multivariable logistic regression was used to examine the association between quitting at Wave 2 and functional as well as risk-minimizing beliefs. Two separate models were run to account for these beliefs at Wave 1 and Wave 2 separately. Additionally, models assessing the mean scores of functional and risk-minimizing beliefs were followed by models that analyze each belief item individually. Similar models were also run for the “planning to quit” outcome at Wave 2. Weights were calculated to adjust for disproportionate sampling respondents in subgroups and longitudinal sampling weights were used for regression analysis. The models also included the covariates: age, sex, education, and income.

## Results

Sample characteristics were calculated using unweighted data and are reported in Table 1. The analytic sample comprised of smokers who responded to both waves of the TCP Survey (*n* = 1112). Of these exclusive smokers at Wave 1, 962 reported still smoking at Wave 2, 36 initiated mixed use, 36 switched to smokeless tobacco use, and 78 respondents quit smoking. Mixed use indicates use of a smoked as well as a smokeless tobacco product. At baseline, mean age was 44 years (SD = 14.17), 97% were male, 66% were aged 25–54 years, and 67% resided in urban areas (Table 1). Overall, 55% of the sample reported having low education level and 82% reported low or moderate income.

**Table 1:** Respondent’s baseline demographic characteristics and smoking tobacco use behaviors^

	**Bihar**	**West Bengal**	**Madhya Pradesh**	**Maharashtra**	**TOTAL**
**N**	111	581	302	118	1112
**Age (M, SD)**	41.8 (17.2)	42.9 (13.6)	45.8 (14.1)	47.5 (12.8)	44.1 (14.2)
**Age (in years) (%)**					
15–17	4 (3.6%)	2 (0.3%)	2 (0.7%)	1 (0.9%)	9 (0.8%)
18–24	18 (16.2%)	34 (5.9%)	22 (7.3%)	5 (4.2%)	79 (7.1%)
25–39	31 (27.9%)	212 (36.5%)	62 (20.5%)	23 (19.5%)	328 (29.5%)
40–54	28 (25.2%)	199 (34.3%)	127 (42.1%)	44 (37.3%)	398 (35.8%)
55 +	30 (27.0%)	134 (23.1%)	89 (29.5%)	45 (38.1%)	298 (26.8%)
**Gender (N, %)**					
Male	87 (78.4%)	573 (98.6%)	302 (100%)	116 (98.3%)	1078 (96.9%)
Female	24 (21.6%)	8 (1.4%)	–	2 (1.7%)	34 (3.1%)
**Urban/Rural (N, %)**					
Urban	85 (76.6%)	418 (71.9%)	160 (53.0%)	84 (71.2%)	747 (67.2%)
Rural	26 (23.4%)	163 (28.1%)	142 (47.0%)	34 (28.8%)	365 (32.8%)
**Education (N, %)**					
Low	44 (39.6%)	294 (51.0%)	211 (69.9%)	58 (49.2%)	607 (54.6%)
Moderate	24 (21.6%)	165 (28.7%)	63 (20.9%)	54 (45.8%)	306 (27.5%)
High	43 (38.7%)	117 (20.3%)	28 (9.3%)	6 (5.1%)	194 (17.5%)
–	–	5 (0.9%)	–	–	5 (0.5%)
**Income (N, %)**					
Low	24 (21.6%)	230 (39.6%)	91 (30.1%)	16 (13.6%)	361 (32.5%)
Moderate	52 (46.9%)	249 (42.9%)	169 (56.0%)	79 (67.0%)	549 (49.4%)
High	32 (28.8%)	95 (16.4%)	30 (9.9%)	18 (15.3%)	175 (15.7%)
Not stated	3 (2.7%)	7 (1.2%)	12 (4.0%)	5 (4.2%)	27 (2.4%)
**Use Frequency (Cigarettes) (N, %)**					
Daily	66 (59.5%)	358 (61.6%)	85 (28.2%)	63 (53.4%)	572 (51.4%)
Less than daily	15 (13.5%)	35 (6.0%)	16 (5.3%)	8 (6.8%)	74 (6.7%)
Less than weekly	4 (3.6%)	47 (8.1%)	4 (1.3%)	2 (1.7%)	57 (5.1%)
–	26 (23.4%)	141 (24.3%)	197 (65.2%)	45 (38.1%)	409 (36.8%)
**Use Frequency (Bidis) (N, %)**					
Daily	15 (13.5%)	313 (53.9%)	224 (74.2%)	59 (50.0%)	611 (55.0%)
Less than daily	1 (0.9%)	11 (1.9%)	5 (1.7%)	4 (3.4%)	21 (1.9%)
Less than weekly	–	17 (2.9%)	–	–	17 (1.5%)
–	95 (85.6%)	240 (41.3%)	73 (24.2%)	55 (46.6%)	463 (41.6%)

^^^The sample characteristics are calculated using unweighted data

– refers to missing data

The functional beliefs were held by 44% to 66% of the respondents (F1 = 58%, F2 = 66%, and F3 = 44%) and risk-minimizing beliefs were held by 10% to 45% (R1 = 10%, R2 = 22%, and R3 = 45%) respondents at Wave 1. At Wave 2, the participants holding functional beliefs ranged from 44 to 61% (F1 = 60%, F2 = 61%, and F3 = 44%) and risk-minimizing beliefs were 14% to 42% (R1 = 14%, R2 = 23%, and R3 = 42%). As evident, the functional beliefs “you enjoy smoking tobacco too much to give it up” and “smoking tobacco calms you down when you are stressed or upset” were held by most respondents (about 60%). Overall, the risk-minimizing beliefs were held by far fewer respondents with least number of people (10%) believing that “the medical evidence that smoking is harmful is exaggerated” and about 40% believing that “smoking is no more risky than lots of other things that people do”.

**Fig. 1:**
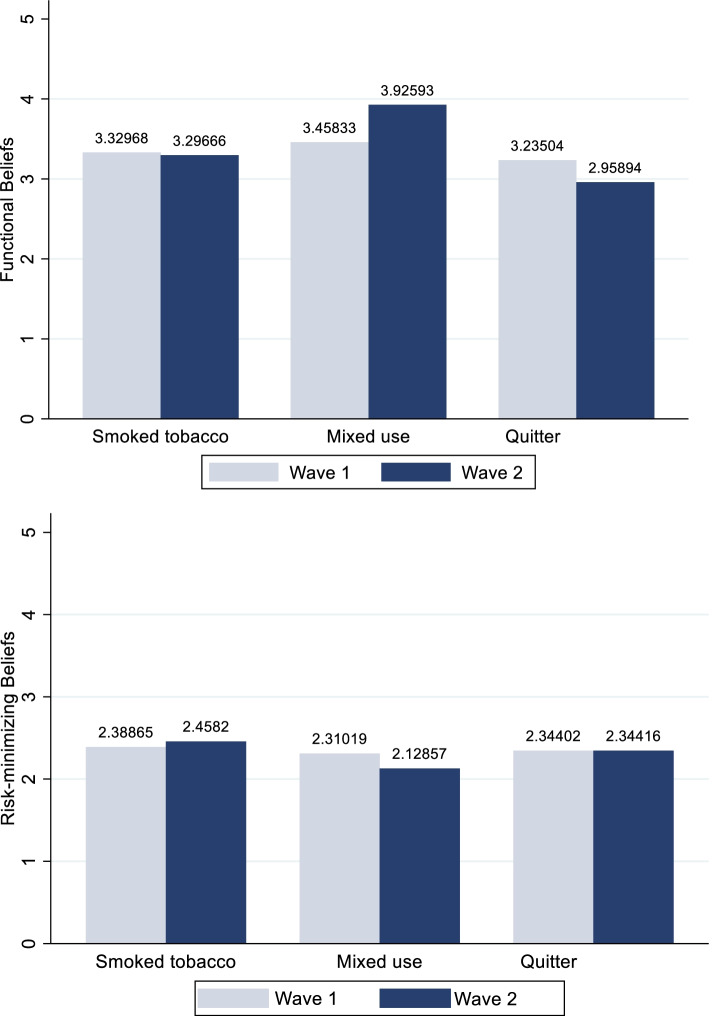
Functional and risk-minimizing beliefs of smokers at Wave 1 and continued smokers, mixed tobacco users and quitter at Wave 2

The mean scores of the beliefs at baseline (with higher scores representing greater agreement) were higher for functional beliefs (F1 = 3.4 (SD = 1.1), F2 = 3.6 (SD = 1.1), and F3 = 3.0 (SD = 1.3) when compared to risk-minimizing beliefs (R1 = 1.9 (SD = 1.0), R2 = 2.4 (SD = 1.1), and R3 = 3.0 (SD = 1.2) (see Table 2). These mean scores did not change significantly for continued smoked tobacco users between the two waves. However, the mean functional belief “you enjoy smoking tobacco too much to give it up” increased significantly for those who transitioned from smoking at Wave 1 into mixed tobacco use at Wave 2 from 3.37 to 4.13 (*p* = 0.004). Smokers at Wave 1 who quit at follow-up had a significant decline in the individual functional beliefs at Wave 2 (*p* < 0.05). The risk-minimizing belief “smoking is no more risky than lots of other things that people do” also declined significantly among those who quit at Wave 2 (*p* = 0.01). There was no significant change in risk-minimizing beliefs among those who transitioned from smoking at Wave 1 to mixed tobacco use at Wave 2. These results show that smokers are more likely to adjust their beliefs according to their changing smoking status, though some beliefs alter more significantly than others (see Fig. 1).

**Table 2:** Summary of smoking-related psychosocial functional and risk-minimizing beliefs^a^

	Wave 1 Smoked Tobacco Users	Wave 2 Smoked Tobacco Users	Wave 2 Quitters	Wave 2^b^ Mixed Tobacco Users
N	1112	962	78	36
**FUNCTIONAL BELIEFS (M, SD)**				
F1. You enjoy smoking too much to give it up	3.4 (1.1) (*n* = 1091)	3.5 (1.2) (*n* = 940)	3.5 (1.2) (*n* = 67)	4.1 (1.0) (*n* = 36)
F2. Smoking calms you down when you are stressed or upset	3.6 (1.1) (*n* = 1098)	3.5 (1.2) (*n* = 939)	3.0 (1.2) (*n* = 68)	4.2 (1.1) (*n* = 35)
F3. Smoking is an important part of your life	3.0 (1.3) (*n* = 1096)	3.0 (1.4) (*n* = 940)	2.4 (1.3) (*n* = 69)	3.4 (1.4) (*n* = 33)
**RISK-MINIMIZING BELIEFS**				
R1. Medical evidence that smoking is harmful is exaggerated	1.9 (1.0) (*n* = 1013)	2.0 (1.1) (*n* = 871)	2.0 (1.1) (*n* = 77)	1.3 (0.7) (*n* = 33)
R2. Everyboy has got to die of something, so why not enjoy yourself and smoke	2.4 (1.1) (*n* = 1045)	2.4 (1.2) (*n* = 890)	NA	2.4 (1.5) (*n* = 31)
R3.Smoking is no more risky than lots of other things people do	3.0 (1.2) (*n* = 970)	3.0 (1.3) (*n* = 872)	2.7 (1.3) (*n* = 76)	3.2 (1.2) (*n* = 26)

^a^Range for beliefs = 1 to 5

^b^Functional beliefs and risk-minimizing beliefs for smokers who transitioned to smokeless tobacco use are not reported as they are pertinent to smoking behaviors only. Similarly, R2. was not measured for quitters and therefore, not reported here

Weighted logistic regression was conducted to assess the association of covariates, functional and risk-minimizing beliefs for two key outcomes: quitting and intentions to quit at Wave 2. The beliefs at both waves were analyzed in separate models (Table 3) and the individual belief items at both waves were analyzed as well (Table 4). Among the covariates, odds of quitting were three times for those in the high-income category when compared to the low-income group (*p* < 0.05). The functional beliefs at Wave 2 were negatively significantly associated with quitting at Wave 2 (OR = 0.63, SE = 0.10, *p* = 0.01). Among the functional beliefs, decline in beliefs “smoking tobacco calms you down when you are stressed or upset” (OR = 0.70, SE = 0.10, *p* = 0.02) and “smoking is an important part of your life” (OR = 0.72, SE = 0.09, *p* = 0.01) were significantly associated with quitting at Wave 2. For those who did not quit at Wave 2 but expressed intentions to quit in the future, there was a marginally significant association with risk-minimizing beliefs at Wave 1 (OR = 1.39, SE = 0.22, *p* = 0.04), primarily driven by the belief that “smoking tobacco is no more risky than lots of other things people do” (OR = 1.45, SE = 0.19, *p* = 0.007). The individual beliefs associated with intentions to quit were the functional beliefs (W2) “enjoy smoking tobacco too much to give it up” (OR = 0.63, SE = 0.08, *p* = 0.002) and “smoking is an important part of your life” (OR = 1.38, SE = 0.17, *p* = 0.02, and risk-minimizing belief (W2) “smoking tobacco is no more risky than lots of other things people do” (OR = 0.82, SE = 0.07, *p* = 0.03).

**Table 3:** Odds Ratios from weighted logistic regression of quitting and plan to quit at Wave 2

	**Quit Smoking**			**Plan to Quit Smoking**		
	OR	SE	*p*-value	OR	SE	*p*-value
		*N* = 859			*N* = 933	
Intercept	0.07	0.09	0.05	**0.01**	**0.01**	0.001**
Age	0.99	0.01	0.95	1.01	0.01	0.29
Sex						
Male	0.86	0.75	0.87	2.28	2.47	0.45
Income						
Moderate	2.07	0.83	0.08	1.87	0.96	0.23
High	**3.55**	**1.57**	0.01*	1.72	1.31	0.48
Education						
Moderate	1.20	0.44	0.61	1.57	0.48	0.14
High	0.93	0.33	0.83	1.46	0.89	0.54
Functional Beliefs (Wave 1)	0.89	0.14	0.44	1.21	0.17	0.19
Risk Minimizing Beliefs (Wave 1)	0.92	0.18	0.66	**1.39**	**0.22**	0.04*
		*N* = 1037			*N* = 923	
Intercept	0.06	0.08	0.05	0.08	0.12	0.11
Age	1.00	0.01	0.75	1.01	0.01	0.23
Sex						
Male	1.61	1.53	0.62	2.17	2.35	0.48
Income						
Moderate	2.48	1.12	0.05	1.68	0.88	0.33
High	**3.97**	**2.03**	0.01**	1.67	1.28	0.51
Education						
Moderate	1.34	0.55	0.48	1.39	0.43	0.30
High	0.99	0.40	0.99	1.35	0.81	0.62
Functional Beliefs (Wave 2)	**0.63**	**0.10**	0.01**	0.77	0.17	0.24
Risk Minimizing Beliefs (Wave 2)	1.03	0.19	0.85	0.73	0.14	0.10

Two models were run. First one with functional and risk-minimizing beliefs at Wave 1 taken together and the second one with these beliefs at Wave 2 taken together

**Table 4:** Odds Ratios from weighted logistic regression of quitting and plans to quit at Wave 2 using individual belief measures

Variable	**Quit Smoking**			**Plan to Quit Smoking**		
	OR	SE	*p*-value	OR	SE	*p*-value
		*N* = 859			*N* = 731	
Intercept	0.12	0.16	0.13	0.01	0.01	0.003
Age	0.99	0.01	0.92	1.01	0.01	0.31
Sex						
Male	0.47	0.35	0.32	1.37	1.71	0.80
Income						
Moderate	2.37	1.15	0.08	1.99	1.05	0.20
High	**3.98**	**2.27**	0.02*	1.82	1.47	0.47
Education						
Moderate	1.24	0.50	0.60	1.78	0.60	0.09
High	0.91	0.38	0.83	1.31	0.87	0.69
**Functional Beliefs (Wave 1)**						
F1. You enjoy smoking						
too much to give it up	1.13	0.17	0.43	0.93	0.12	0.59
F2. Smoking tobacco calms						
you down when stressed or upset	0.87	0.14	0.41	1.13	0.15	0.34
F3. Smoking is an						
important part of your life	0.85	0.12	0.25	1.15	0.15	0.28
**Risk Minimizing Beliefs**						
R1. Medical evidence that						
is harmful is exaggerated	0.92	0.12	0.51	1.10	0.25	0.67
R2. Everybody has got to die of						
something so why not smoke	0.85	0.13	0.30	0.89	0.12	0.39
R3. Smoking is no more						
risky than lots of other things	1.19	0.14	0.14	**1.45**	**0.19**	0.007**
		*N* = 877*			*N* = 740*	
Intercept	0.06	0.08	0.03	0.30	0.32	0.26
Age	1.01	0.01	0.66	1.01	0.01	0.47
Sex						
Male	1.66	1.29	0.52	–	–	–
Income						
Moderate	2.04	0.92	0.12	1.57	0.75	0.36
High	**3.02**	**1.52**	0.03*	1.80	1.14	0.36
Education						
Moderate	1.23	0.52	0.62	1.29	0.39	0.41
High	0.94	0.38	0.87	0.99	0.59	0.99
**Functional Beliefs (Wave 2)**						
F1. You enjoy smoking						
too much to give it up	1.36	0.21	0.06	**0.63**	**0.08**	0.002**
F2. Smoking tobacco calms						
you down when stressed or upset	**0.70**	**0.10**	0.02*	**1.38**	**0.17**	0.02*
F3. Smoking is an						
important part of your life	**0.72**	**0.09**	0.01*	0.88	0.13	0.38
**Risk Minimizing Belief**						
R1. Medical evidence that						
is harmful is exaggerated	1.06	0.13	0.62	0.91	0.13	0.49
R2. Everybody has got to die of						
something so why not smoke	–	–	–	0.89	0.12	0.40
R3. Smoking is no more						
risky than lots of other things	0.87	0.12	0.29	**0.82**	**0.07**	0.03*

## Discussion

The main aim of this study was to analyze the functional and risk-minimizing beliefs and their associations with quitting behavior and intentions at Wave 2 among exclusively smoking tobacco users at baseline. Our findings show that the pattern of belief change, particularly among functional beliefs is consistent with dissonance reduction and evidence from previous studies. These beliefs stay consistent over time among continued smokers but become stronger among those who transition to mixed tobacco use at Wave 2 and become weaker among those who quit. The results, among this population show a greater magnitude of change among functional, but not risk-minimizing beliefs overall.

The psychosocial beliefs assessed were *functional beliefs* which reinforce the role of smoking in one’s life and *risk-minimizing* beliefs which tend to reduce the perception of harm caused by tobacco use. Overall, there was a greater percentage of respondents who agreed with functional beliefs at both waves. The functional beliefs “you enjoy smoking too much to give it up” and “smoking calms you down when you are stressed or upset” were held by about 60% respondents at both waves with 44% agreeing that “smoking is an important part of your life”. As Fotuhi et al. (2013) concluded, functional beliefs may be less susceptible to encounter resistance as they are not easy to challenge using counterarguments and rationale. In comparison, the risk-minimizing beliefs were held by fewer smokers with highest agreement (44%) for “smoking is no more risky than lots of other things people do” at both waves. About 22% respondents held the belief that “everybody has got to die of something so, why not enjoy yourself and smoke” and the least supported belief was “medical evidence that smoking is harmful is exaggerated” held by 10% smokers. These beliefs are considered “weak beliefs” as they might be susceptible to being easily changed [16, 24]. An overall lower agreement with risk-minimizing justifications is a positive sign overall and bolsters the support for policies (such as graphic warning labels) and education campaigns to highlight harms of smoking in India.

Among the smokers that quit successfully at Wave 2, there was no change in the risk-minimizing beliefs as they were quite low to begin with. There was, however, a reduction in functional beliefs among those who quit. The levels of functional beliefs were similar at baseline for smokers but significantly changed as they transitioned to mixed tobacco use or quitting at follow-up; the levels increased among mixed tobacco users and declined among quitters, in concordance with their smoking behaviors. Regression analysis shows a significant negative association of functional beliefs at Wave 2 with quitting smoking at Wave 2; these beliefs at Wave 1, however, had no significant association with quitting at Wave 2. Therefore, those who quit are more likely to express reduction in functional beliefs over time. Evidence suggests that functional beliefs play a crucial role in early periods of quitting, wherein highly dependent smokers and those holding strong functional beliefs are at greater risk of relapse [25]. Future cessation efforts and tobacco control campaigns can target these beliefs to inoculate smokers against tobacco marketing that highlights the functional aspects of smoking (concentration, calmness, weight loss etc.) and boost self-efficacy in quitting overall.

Risk-minimizing beliefs at Wave 1 were marginally significantly associated with intentions to quit at Wave 2, driven by the belief “smoking tobacco is no more risky than lots of other things people do”. However, this association was positive which seems counterintuitive. Beliefs at Wave 2, that were negatively associated with intentions to quit were F1 (You enjoy smoking too much to give it up) and R3 (Smoking is no more risky than lots of other things that people do) whereas F2 (Smoking calms you down when you are stressed or upset) was positively associated. Given the incoherent patterns of these associations with intentions to quit, it is worthy of further investigation.

The association between health beliefs and smoking behavior may differ based on sociocultural factors and norms [16, 26]. These findings, particularly ones tracking patterns of beliefs and their association with quitting at Wave 2, highlight the key beliefs that drive smoking behaviors and provide evidence from a low-middle income country context. It adds to the larger literature in tobacco research that seeks to determine if these phenomena are culturally universal and whether these associations differ by countries. The study analyzing the association between smoking rationalizations and intention to quit smoking from China, utilized a smoking rationalization scale developed specifically from a population-based sample of Chinese male smokers within the socio-cultural context [19]. Since these beliefs are driven by culture, tobacco marketing efforts, and regulatory environments, more research is needed to develop and evaluate the reliability of smoking belief measures in different contexts.

These findings should be interpreted in the light of a few limitations. First, the data is self-reported at two different time points which may be subject to recall and/or social desirability bias. Second, the smoking tobacco sub-sample selected for this study comprised cigarette and bidi users at Wave 1. It is possible that the beliefs held by users of either product are distinct which make them prefer a filtered cigarette over the unfiltered bidis. This could be investigated in subsequent studies alongside assessment of beliefs among smokeless tobacco users and vulnerable groups. Future studies focusing on different forms of tobacco use and populations (such as rural vs urban) can aid in addressing tobacco use related disparities. This study utilized two waves of data from the cohort of smokers which provides more information than cross-sectional data, but future waves of data may illuminate patterns of beliefs among those who continued smoking, relapsed quitters, or those who successfully quit. Lastly, we assessed the patterns and associations of two key types of beliefs based on prominent tobacco literature, but there is a wide array of psychosocial beliefs surrounding tobacco use that may be worthwhile to analyze in different cultural contexts, even if they were not found to be influential in some countries.

## Conclusion and implications

The study advances our understanding of the role that self-exempting beliefs and justifications play in smoking tobacco use and cessation, demonstrating that in the vastly different cultural context of India, strategies (whether conscious or not) to reduce dissonance among smokers may be quite similar to those among smokers in high-income countries. In both India and Western countries, these beliefs seem to play an integral role in dissonance reduction and undergo shifts with one’s own tobacco consumption behavior. A broader understanding of these belief patterns, especially in different regulatory and cultural contexts can be influential in developing effective tobacco control programs and policies.

## Supplementary Information

**Additional file 1.**

## Data Availability

The data are jointly owned by a third party in each country that collaborates with the International Tobacco Control Policy Evaluation (ITC) Project. Data from the ITC Project are available to approved researchers 2 years after the date of issuance of cleaned data sets by the ITC Data Management Centre. Researchers interested in using ITC data are required to apply for approval by submitting an International Tobacco Control Data Repository (ITCDR) request application and subsequently to sign an ITCDR Data Usage Agreement. The criteria for data usage approval and the contents of the Data Usage Agreement are described online (http://www.itcproject.org). The authors of this paper obtained the data following this procedure. This is to confirm that others would be able to access these data in the same manner as the authors. The authors did not have any special access privileges that others would not have. The data that support the findings of this study are available from the ITC Project, but restrictions apply to the availability of these data, which were used under license for the current study, and so are not publicly available. Data are however available from the authors (ackquah@uwaterloo.ca) upon reasonable request and with permission of the ITC Project team.

## Abbreviations

HIC: High Income Countries
LMIC: Low- and Middle- Income Countries
STATA: Software for Statistics and Data Science developed by StataCorp
TCP IN Survey: Tobacco Control Policy India Survey
