# Supplementary material for: Smoking-related psychosocial beliefs and justifications among smokers in India: Findings from Tobacco Control Policy (TCP) India Surveys
Source: BMC Public Health. 2022 Sep 13;22:1738. doi: 10.1186/s12889-022-14112-w (PMC9472368; doi:10.1186/s12889-022-14112-w)
Supplement: Supplementary file 1 — Additional file 1. [file 12889_2022_14112_MOESM1_ESM.docx]

Description of analytic sample of smokers using Wave 1 and Wave 2 of TCP India survey data
